# Myocardial protective effect and transcriptome profiling of Naoxintong on cardiomyopathy in zebrafish

**DOI:** 10.1186/s13020-021-00532-0

**Published:** 2021-11-14

**Authors:** Mengyan Hu, Peirong Liu, Shuxian Lu, Zhihao Wang, Zhaojie Lyu, Hongkai Liu, Yuhong Sun, Feng Liu, Jing Tian

**Affiliations:** 1 Western China Zebrafish Research Center for Human Diseases and Drug Screening, The College of Life Sciences, Northwest University, Xi’an, 710069 China; 2 Key Laboratory of Resource Biology and Biotechnology in Western China, Ministry of Education, School of Medicine, Northwest University, Xi’an, 710069 China; 3 Shaanxi Buchang Pharmaceutical Co. Ltd., Xi’an, 710075 China; 4 Shaanxi Institute of International Trade and Commence, Xi’an, 712046 China

**Keywords:** Cardiomyopathy, Naoxintong (NXT), Zebrafish, Transcriptome, HEG1

## Abstract

**Background:**

Cardiomyopathy is a kind of cardiovascular diseases, which makes it more difficult for the heart to pump blood to other parts of the body, eventually leading to heart failure. Naoxintong (NXT), as a traditional Chinese Medicine (TCM) preparation, is widely used in the treatment of cardiovascular diseases, including cardiomyopathy, while its underlying mechanism has not been fully elucidated. The purpose of this study is to investigate the therapeutic effect of NXT on cardiomyopathy and its molecular mechanism in zebrafish model.

**Methods:**

The zebrafish cardiomyopathy model was established using terfenadine (TFD) and treated with NXT. The therapeutic effect of NXT on cardiomyopathy was evaluated by measuring the heart rate, the distance between the sinus venosus and bulbus arteriosus (SV-BA), the pericardial area, and the blood flow velocity of zebrafish. Then, the zebrafish hearts were isolated and collected; transcriptome analysis of NXT on cardiomyopathy was investigated. Moreover, the *heg1* mutant of zebrafish congenital cardiomyopathy model was used to further validate the therapeutic effect of NXT on cardiomyopathy. Additionally, UPLC analysis combined with the zebrafish model investigation was performed to identify the bioactive components of NXT.

**Results:**

In the TFD-induced zebrafish cardiomyopathy model, NXT treatment could significantly restore the cardiovascular malformations caused by cardiac dysfunction. Transcriptome and bioinformatics analyses of the TFD and TFD  +  NXT treated zebrafish developing hearts revealed that the differentially expressed genes were highly enriched in biological processes such as cardiac muscle contraction and heart development. As a cardiac development protein associated with cardiomyopathy, HEG1 had been identified as one of the important targets of NXT in the treatment of cardiomyopathy. The cardiovascular abnormalities of zebrafish *heg1* mutant could be recovered significantly from NXT treatment, including the expanded atrial cavity and blood stagnation. qRT-PCR analysis further showed that NXT could restore cardiomyopathy phenotype in zebrafish through HEG1-CCM signaling. Among the seven components identified in NXT, paeoniflorin (PF) and salvianolic acid B (Sal B) were considered to be the main bioactive ones with myocardial protection.

**Conclusion:**

NXT presented myocardial protective effect and could restore myocardial injury and cardiac dysfunction in zebrafish; the action mechanism was involved in HEG1-CCM signaling.

**Supplementary Information:**

The online version contains supplementary material available at 10.1186/s13020-021-00532-0.

## Background

Cardiomyopathy, a kind of cardiovascular diseases (CVD), is a mechanical and cardiac dysfunction of the heart muscle, which can lead to cardiac death or heart failure [1]**.** The most typical feature of cardiomyopathy is abnormal myocardial structure and systolic function. Because the regulation of myocardial contractility directly affects the myocardium and the general cardiovascular system, cardiomyopathy is often accompanied by ventricular enlargement caused by intraventricular blood stagnation, resulting in cardiac pumping dysfunction [2]. Cardiomyopathies are classified according to phenotype as hypertrophic cardiomyopathy (HCM), dilated cardiomyopathy (DCM), restrictive cardiomyopathy (RCM), arrhythmogenic right ventricular cardiomyopathy (ARVC), and unclassified cardiomyopathies [3]. Among them, DCM, characterized by cardiac enlargement and loss of systolic function, has the highest prevalence among all cardiomyopathies [4].

Zebrafish (*Danio rerio*) has become a powerful tool for the study of human diseases. Some or most human pathological phenomena can be observed in zebrafish. The heart is the first functioning organ to be formed during zebrafish embryogenesis [5, 6]. Although the structure of the zebrafish embryonic heart is simpler than that of mammals, with only two chambers, atrium and ventricle, the morphological processes and the regulatory molecular pathways of the zebrafish heart formation are similar to those of mammalians. Zebrafish embryos and larvae are small and transparent, and can be used to observed organ formation and related biological processes, especially cardiovascular development, with high resolution in vivo. Furthermore, with the benefit from the transgenic lines and genetic manipulation tools available such as CRISPR-Cas9 technology [7], zebrafish has become particularly useful in cardiogenesis. There are several zebrafish disease models generated and applied not only for the research of related diseases, but also for the related drug screening and therapeutic efficacy analysis [8]. Gu et al. developed a dilated cardiomyopathy model in zebrafish larvae with terfenadine (TFD). It is characterized by atria and ventricle swelling, decreased blood circulation and heart rate, which was a good model of non-ischemic heart failure [9]. In our previous study, by specific knocking out a heart development protein heg1 using CRISPR/Cas9 technology, we established a zebrafish congenital dilated cardiomyopathy model, *heg1*^*△25*^ mutant. Zebrafish *heg1*^*△25*^ mutant showed very obvious pericardial swelling, pericardial edema, and heart rate slowing, which were similar to those in patients with heart failure [10]. *heg1*^*△25*^ mutant was also used to verify the therapeutic effect of Traditional Chinese Medicine (TCM) and monomers in the treatment of cardiovascular diseases [10].

In recent years, TCM has been more and more widely used in the treatment of cardiovascular diseases [11]. Naoxintong (NXT) is a well-known prescribed TCM, which is commonly used for the prevention and treatment of cardiovascular and cerebrovascular diseases. It has been clinically used for more than two decades [12]. NXT consists of 16 herbs, including *Astragali Radix* (Huangqi), *Salviae miltiorrhizae Radix et Rhizoma *(Danshen), *Paeoniae Radix Rubra *(Chishao), *Angelicae Sinensis Radix *(Danggui), *Cinnamomi Ramulus* (Guizhi), Persicae Semen (Taoren), *Chuanxiong Rhizoma* (Chuanxiong), *Spatholobi Stem* (Jixueteng), *Mori Ramulus *(Sangzhi), *Carthami flos* (Honghua), *Achyranthis* (Niuxi), *Olibanum* (Ruxaing), *Myrrha Achyranthis* (Moyao), *Pheretima* (Dilong), *Scorpio* (Quanxie), *Hirudo* (Shuizhi) [13]. Recent studies have shown that NXT plays a cardioprotective role by affecting inflammation, apoptosis, oxidative stress, neovascularization, insulin sensitivity and lipid/glucose metabolism, as well as restoring ischemia injury [14, 15]. In the analysis of blood samples from 69 patients with type 2 diabetes mellitus (T2DM) treated with NXT, it was found that NXT was able to restore the effects of HDL on the proliferation, apoptosis and angiogenesis of human umbilical vein endothelial cells [16]. In the rat coronary microembolism (CME) model, NXT significantly reduced the number of CME and cardiomyocyte apoptosis [17]. In the mouse model of myocardial ischemia/reperfusion (I/R) injury, Wang et al. found that NXT improved ventricular function and reduced infarct size after injury by inhibiting NLRP3 inflammatory vesicle activation [18]. In H_2_O_2_-induced endothelial cell injury, NXT aqueous extract could reduce apoptosis and autophagy by inhibiting the activation of caspase-3/PARP-1 signaling pathway [19]. In terms of vascular protection, NXT could improve ischemia–reperfusion injury as well as lipid metabolism, and also had the effect of inhibiting vascular inflammation and protecting the vascular endothelium [20]. Our previous study also revealed that NXT could repair cellular hypoxia and glucose damage in cell OGD/R model, and improve the symptoms of cardiac/vascular ischemia by regulating COX2-VEGF/NFκB signaling in the zebrafish thrombosis model [15]. However, the underlying molecular mechanism of NXT in the treatment of myocardial injury has not been fully elucidated, and still need to be further studied.

In this study, TFD-induced and congenital zebrafish dilated cardiomyopathy models were applied to evaluate the myocardial protective effect of NXT on cardiomyopathy. The potential molecular mechanism of its action was investigated through transcriptome profiling analysis.

## Materials and methods

### Ethics statement

The zebrafish experiments were conducted according to the ethical guidelines of Northwest University. All experimental protocols were approved by the Experimental Animal Management and Ethics Committee of Northwest University, and the ethical code was NWU-AWC-20190103Z.

### Preparation of NXT extract

Naoxintong Capsule (NXT) was kindly provided by Shaanxi Buchang Pharmaceuticals. The extraction process of NXT was carried out by water extraction. In brief, 10 g of NXT powder was soaked in 100 ml distilled water for 30 min, followed by ultrasonic (250 W, 50 kHz) for 30 min, and evaporated to 10 ml and filtered; the stock concentration is 1 g/ml and kept at − 80 °C.

### Zebrafish maintenance and embryos handling

All adult zebrafish were maintained in zebrafish culture system with a light cycle of 14 h light/10 h dark. Zebrafish embryos were produced by natural crosses and cultured in egg water at 28.5 °C [21]. The following strains were used: AB wild-type (wt) strain, transgenic *Tg*(*cmlc2:eGFP*) strain expressing enhanced green fluorescent protein (GFP) in cardiomyocytes [22], *Tg*(*flk1:eGFP*) strain expressing enhanced green fluorescent protein (GFP) in blood vasculature [23], *heg1*^*∆25*^ mutant, *Tg*(*heg1*^*∆25*^; *cmlc2:eGFP*) transgenic line, and *Tg*(*heg1*^*∆25*^;* flk1:eGFP*) transgenic line [10].

### Drug treatment

Zebrafish *Tg*(*cmlc2:eGFP*) embryos were incubated in egg water until 48 h-post-fertilization (hpf). Four experimental groups were set up as: control group, TFD group, TFD  +  NXT group and NXT group. Embryos were collected in 6-well plates with 15 embryos per well and three replicates were set for each group. The control group was treated with 0.1% dimethyl sulfoxide (DMSO) for 48 h; the NXT group was treated with NXT at a concentration of 600 mg/L for 48 h; the TFD group was treated with 15 µM TFD (Solarbio, China) for 48 h to obtain zebrafish cardiomyopathy model [9]. For TFD  +  NXT group, the embryos were treated with 15 µM TFD for 48 h first, followed by NXT treatment for another 24 h. To optimize the concentrations of NXT, the following doses of NXT were used, 200, 300, 400, 500, 600, 700, 800, 900 and 1000 mg/L. For *heg1* mutants rescue experiment, *heg1* mutant embryos at 48 hpf were collected and placed into 6-well plates. To optimize the concentrations of NXT, 500, 1000, 1500, 2000, 2200, 2400, 2500, 2600, and 2800 mg/L of NXT were used to treat *heg1* mutant embryos for 24 h. The recovery rate of heart rate and blood flow were recorded at the end of the treatment, 45 embryos per group were used for statistics analysis.

### Cardiac morphology analysis

After treatment, embryos were collected at 96 hpf for phenotype analysis. Zebrafish embryos were mounted in 5% methylcellulose. Images were obtained using a Nikon SMZ25 stereomicroscope (Nikon, Japan) for morphology observation. Photographs were analyzed using NIS-Elements BR software (Nikon, Japan) to determine the distance between sinus venosus and bulbus arteriosus (SV-BA) and pericardial area per field of view.

### Heart rate and blood flow rate statistics

The heart beats of embryos per unit of time was counted with a stopwatch and a counter, expressed as beats per minute (BPM). Video imaging of the movement of red blood cells (RBCs) in the posterior cardiac veins (PVCs) of 96 hpf zebrafish embryos was acquired. DanioScope software (Noldus, Netherland) was used to analyze the movement ratio of RBCs based on changes in pixel density, and the results were expressed as the ratio of blood flow pixel values to surrounding tissue.

### Heart isolation of zebrafish embryos

Zebrafish embryonic heart dissection was carried out according to the procedure adapted from Lombardo [24]. Briefly, 200–300 *Tg(cmlc2:eGFP)* zebrafish embryos at 96 hpf from four groups were collected respectively, and placed in 1.5 mL tube on ice. 1 mL L-15/10% FBS medium was added. The embryos pericardial cavity was destructed using a 5 mL syringe with a 20-gauge needle. GFP-positive hearts were separated manually under fluorescence microscope and placed in L-15/10% FBS medium for further study.

### RNA-seq and bioinformatic analysis

After zebrafish embryonic hearts were isolated, total RNA was extracted from four group samples of zebrafish embryonic hearts using Trizol (Life Technologies, USA), including control group, NXT group, TFD group, and TFD  +  NXT group, respectively [25]. Libraries were constructed by using NEBNext Ultra™ RNA Library Prep Kit for Illumina (NEB, USA) and sequenced by Illumina novaseq 6000 platform (NovoTech, China). The clean reads were mapped to reference genome (Danio rerio: NCBI_GRCz11). Differential expressed genes (DGE) analysis of the two groups was performed using the DESeq2 R package (1.10.1). The *p *values were adjusted using the Benjamini and Hochberg's approach for controlling the false discovery rate. Genes with an adjusted *p* value  < 0.001 and |log_2_(Fold change)|≥ 2 were considered as differentially expressed.

The DEGs were subjected to enrichment analyses of Gene Ontology (GO) and KEGG pathways analysis. GO terms and KEGG analysis with corrected *p* value  < 0.05 were considered as significantly enriched.

### Real-time quantitative PCR (qRT-PCR)

For qRT-PCR analysis, total RNA was extracted from whole embryos of zebrafish in each group using TRIzol reagent (Life Technologies, USA). The cDNA was synthesized using the SuperScriptIII (Invitrogen, USA). qRT-PCR detection was conducted by the SYBR FAST Universal qPCR kit (KAPA, Germany) and ViiA 7 Real-Time PCR System (ABI, USA). The reaction system was as follows: 95 °C pre-denaturation for 3 min; 95 °C for 5 s, 60 °C for 30 s, 40 cycles. The quantified values were generated from the average results of three independent experiments with the triplicate PCR runs [21]. Primer sequences are listed (Additional file 1: Table S1).

### Chemical analysis of NXT by UPLC

The composition of NXT was identified using Ultra Performance Liquid Chromatography (UPLC) method. The mixed chemical standards, including mulberroside A (MulA), hydroxysafflor yellow A (HSYA), amygdalin (AMY), paeoniflorin (PF), ferulic acid (FA), calycosin 7-*O*-glucoside (CG), rosmarinic acid (RA), salvianolic acid B (Sal B), calycosin (CAL), formononetin (FN) and tanshinone IIA (Tan IIA) were bought from National Instisutes for Food and Drug Control (China). The separation was achieved using a WATERS ACQUITY UPLC BEH C18 column (2.1 mm × 100 mm, 1.7 μm), with a mobile phase consisting of 0.2% formic acid (phase A) and acetonitrile (phase B) at a flow rate of 0.2 mL/min at a temperature of 35 °C. UV detection at 254 nm was applied. The UPLC fingerprint of NXT was recorded and analyzed by comparing with the references.

Control embryos or TFD-induced embryos were treated with PF (10 μM) or Sal B (10 μM) or PF:SalB (1:1) mixture, respectively. All phenotypical and qRT-PCR analysis were described as above.

### Statistical analysis

Each experiment was conducted at least three times. Data analysis was performed using Graph Pad Prism 5.0 software. All data were presented as means  ±  SD. Statistical evaluation was performed by one-way ANOVA analysis. *p*  <  0.05 was considered as statistically significant.

## Results

### NXT restored TFD-induced cardiomyopathy in zebrafish

**Fig. 1:**
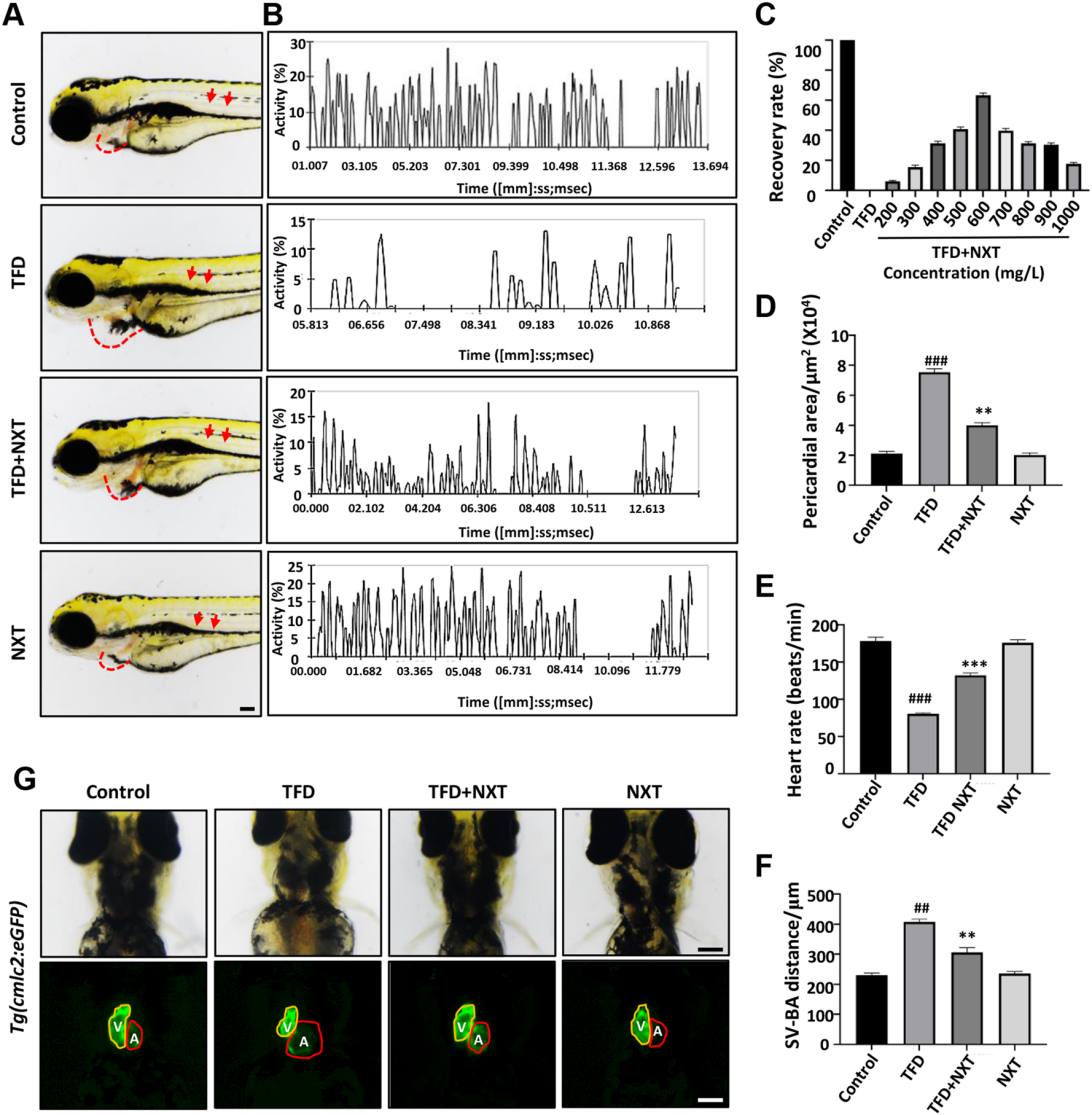
NXT treatment restored TFD-induced cardiomyopathy in zebrafish embryos. **A** Lateral view of four groups of zebrafish embryos at 4 dpf. TFD treated group showing pericardial edema (red dashed line) and slowed blood flow (red arrows). **B** Blood flow motion ratios of four groups of zebrafish embryos based on pixel density changes in RBCs. **C** Optimal recovery concentration of NXT. **D** Pericardial area of four groups of zebrafish larvae (n  =  15 embryos/group). **E** Heart rates of four groups of zebrafish larvae (n  =  15 embryos/group) **F** SV-BA distance of the four groups of zebrafish larvae (n  =  15 embryos/group). **G** The heart morphology of four groups embryos at 96hpf delineated by *Tg*(*cmlc2:GFP*) (ventral view). Note the enlarged ventricles in the TFD treatment group (*V* ventricle; *A* atria). Data are represented mean  ±  standard deviation (SD) from three independent experiments, ^##^*p*  <  0.01, ^###^*p*  < 0.001 vs control group; **p  < 0.01, ***p  < 0.001 vs TFD-induced group (Student’s t test)

To investigate the myocardial protective effect of NXT on cardiomyopathy, we used 15 μM TFD to establish a zebrafish cardiomyopathy model. As shown in Fig. 1, compared with control group, zebrafish in the TFD treated group exhibited severe cardiac malformations, including pericardial edema (Fig. 1A, red dash line), venous congestion (Fig. 1A, red arrow), dramatically blood flow reduction (Fig. 1B), increased pericardial area (Fig. 1D), slow heart rate (Fig. 1E), as well as abnormally increased SV-BA distance (Fig. 1F). The zebrafish cardiomyocyte specific transgenic reporter line *Tg*(*cmlc2:eGFP*), when treated with TFD, the embryo atrium was dilated, with abnormal atrial (A) and ventricular (V) morphology (Fig. 1G). This is similar to the pathophysiology of dilated cardiomyopathy (DCM).

After treatment with different concentrations of NXT (200–1000 mg/L), the cardiomyopathy phenotype induced by TFD was restored to varying degrees. The optimal recovery concentration was 600 mg/L for further experiments (Fig. 1C). Compared with the TFD group, the TFD + NXT group embryos exhibited a 65% decrease in the enlarged pericardial area (Fig. 1A, D), a significant recovery of blood flow (Fig. 1B), a 44.3% reduction in abnormal increased SV-BA distance (Fig. 1F), and a close to normal atrium and ventricle morphology (Fig. 1G). The heart rate of TFD group was reduced to 80  ±  5 BPM compare with the heart rate of 175  ±  5 BPM in the control group, and the heart rate was restored to 132  ±  5 BPM in TFD  +  NXT group (Fig. 1E). Therefore, NXT could significantly restore the phenotype of TFD induced cardiomyopathy in zebrafish.

### Transcriptome analysis of zebrafish cardiomyopathy embryo hearts treated with NXT

**Fig. 2:**
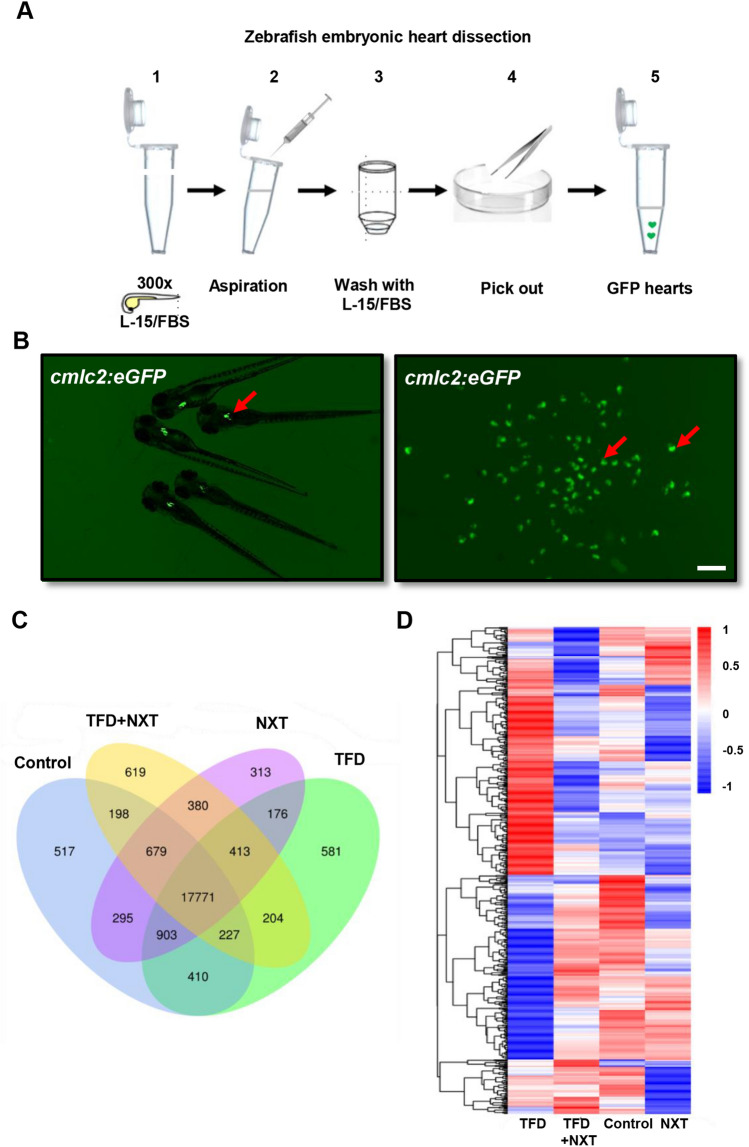
Transcriptome analysis of differentially expressed genes in NXT treated cardiomyopathy zebrafish hearts. **A** A schematic overview of heart isolation in zebrafish embryos. **B** The isolated cardiac tissues from *Tg*(*cmlc2:eGFP*) transgenic fish embryos at 96 hpf. **C** Venn diagram analysis and **D** Heat map representation showing DGEs in control, TFD, TFD  +  NXT and NXT group

In order to identify the molecular targets of NXT in the treatment of cardiomyopathy, *Tg*(*cmlc2:eGFP*) zebrafish embryonic hearts at 96 hpf were isolated from each group, including control, TFD, TFD  +  NXT, and NXT, respectively (Fig. 2A, B). Total RNA was extracted from zebrafish embryonic hearts followed by RNA-Seq analysis. There were 17,771 transcripts expressed in the four transcriptome groups (Fig. 2C). The expression patterns of screened genes were subjected to cluster analysis as shown in Fig. 2D.

**Fig. 3:**
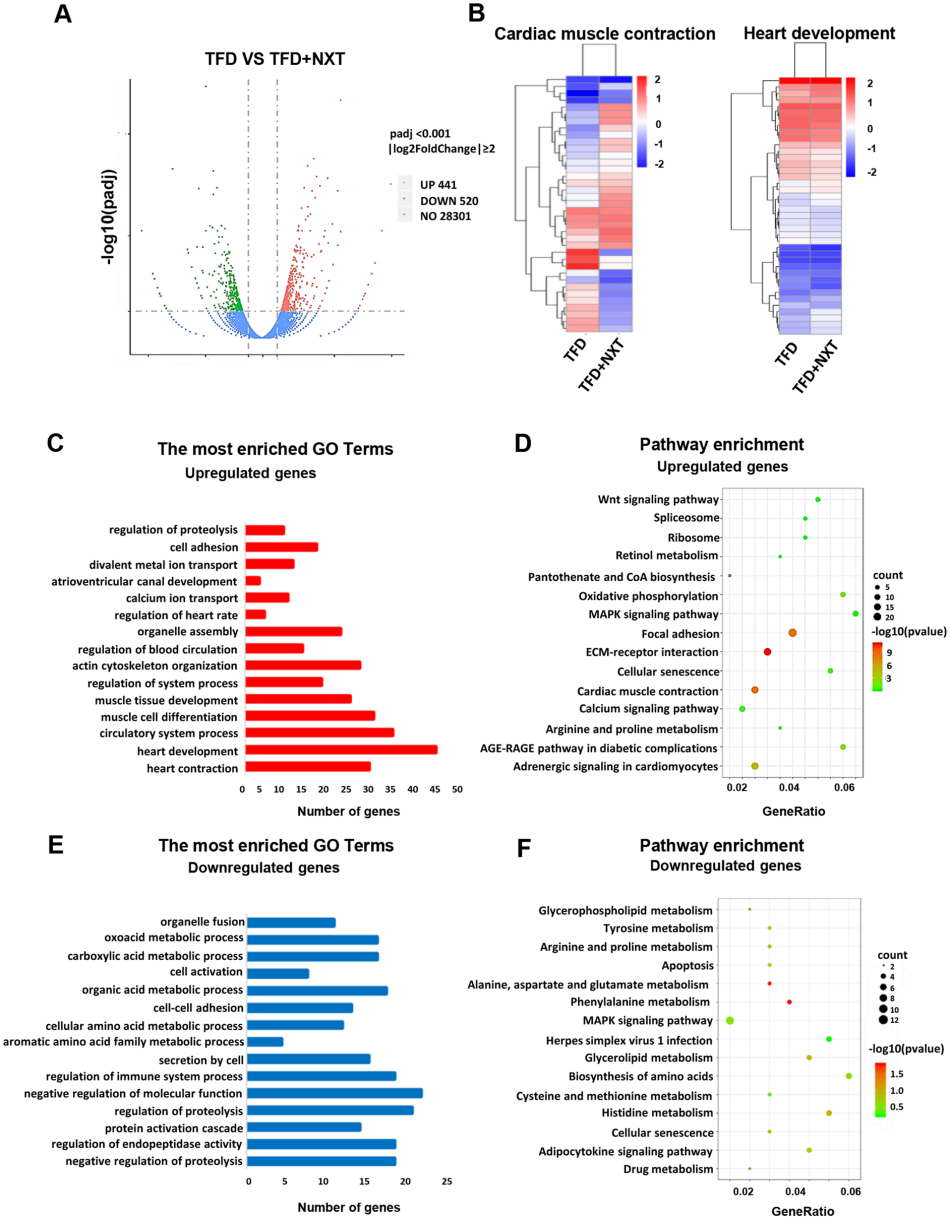
Bioinformatics analysis between TFD and TFD  +  NXT groups. **A** Volcano plots showing a total of 961 DGEs in TFD and TFD  +  NXT groups. **B** A heat-map showed that genes involved in cardiac muscle contraction and heart development. **C** GO term analysis and **D** KEGG pathway analysis for upregulate DEGs in TFD  +  NXT zebrafish embryo hearts compared to TFD group. **E** GO term analysis and **F** KEGG pathway analysis for upregulate DEGs in TFD  +  NXT zebrafish embryo hearts compared to TFD group. The size of the dot indicates the number of genes enriched in individual item. The color of the dot represents the *p* value

After quality filtering, a total of 961 differentially expressed genes (DEGs) was identified in TFD and TFD  +  NXT groups (|log_2_(FoldChange)|≥ 2, *p*  < 0.001). Among these genes, 441 genes were upregulated and 520 genes were downregulated (Fig. 3A, Additional file 2: Table S2). Not surprisingly, a number of genes highly associated with cardiac development were altered, including those involved in cardiac muscle contraction (up-regulated: 27; down-regulated: 12) and heart development (up-regulated: 34; down-regulated: 6) (Fig. 3B).

In order to explore whether this change was related to specific pathways or biological process, we carried out Gene ontology (GO) functional enrichment analysis and KEGG pathway enrichment analysis (Additional file 3: Table S3). It was found that the upregulated genes were mainly enriched in cardiovascular system development, including heart development, blood circulation, and cardiac muscle cell and tissue development (Fig. 3C); the downregulated genes were enriched in regulation of proteolysis, endopeptidase activity, immune system process, and the metabolic process of oxoacid, carboxylic acid, and organic acid (Fig. 3E). KEGG analysis also revealed that the upregulated genes were mainly in cardiac muscle contraction and ECM-receptor interaction pathways (Fig. 3D); the downregulated genes were mainly in MAPK signaling pathway and metabolism pathways of glycerophospholipid and amino acid (Fig. 3F). This finding suggested that the restorative effect of NXT on zebrafish cardiomyopathy might be achieved by influencing key myocardial signaling pathways and the biological process of cardiac development.

### NXT alleviated zebrafish cardiomyopathy by regulating of myocardial and cardiovascular development related genes

**Fig. 4:**
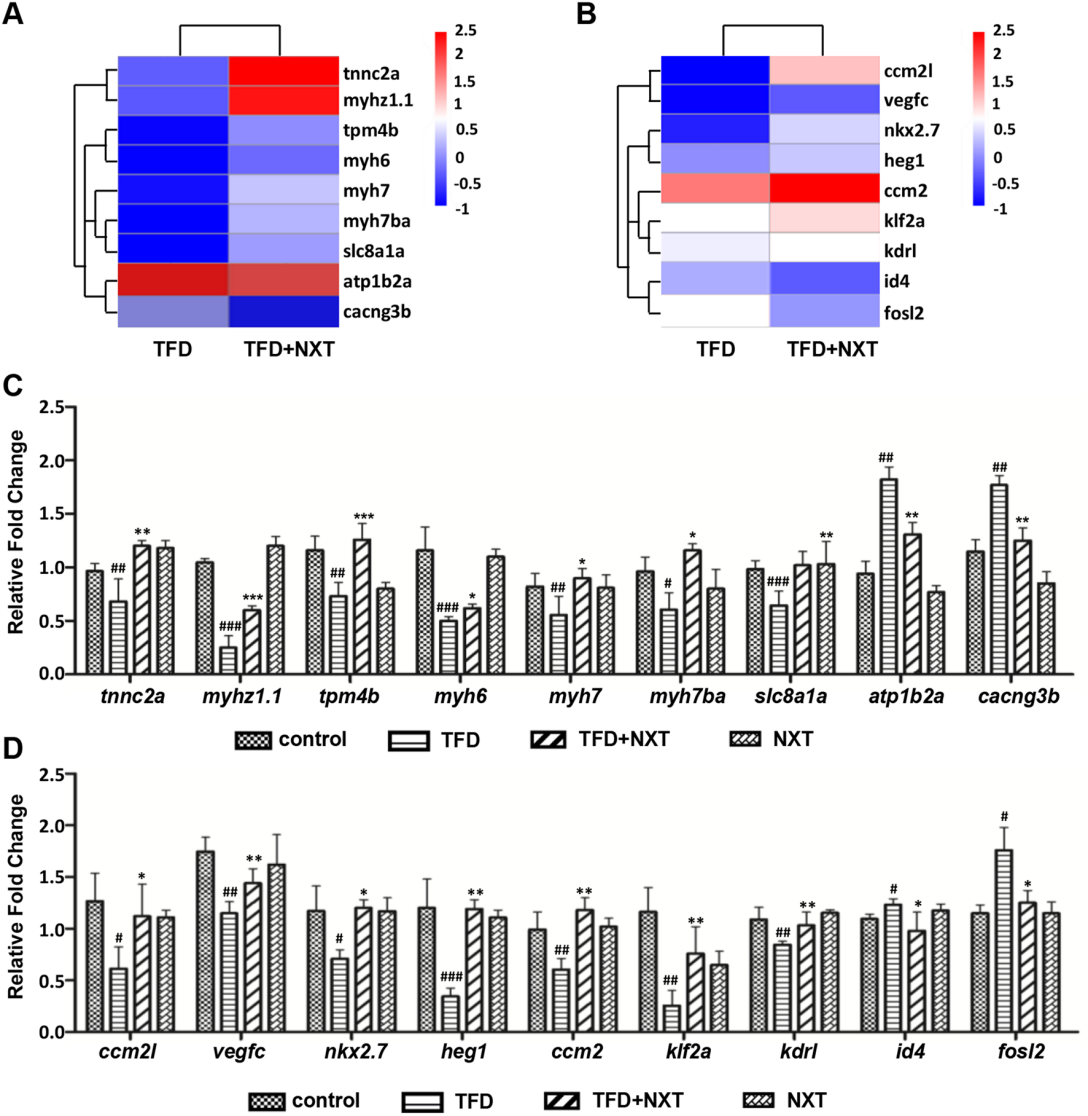
NXT targeting node screening and downstream pathways verification. **A** myocardium related genes and **B** cardiovascular development related genes explored by heat map analysis, and verified by qRT-PCR (**C**, **D**). Data are represented as mean  ±  SD. ^#^*p*  <  0.05, ^##^*p*  <  0.01, ^###^*p*  <  0.001 vs control group; *p  <  0.05, **p  <  0.01, ***p  <  0.001 vs TFD-induced group

Previous studies of human genetics have shown that there are multiple genes related to dilated cardiomyopathy (DCM) [26–29]. Based on RNA-Seq data obtained here from zebrafish heart, we summarized 30 DCM related genes homologous to human in zebrafish (Table 1), including cardiac actin, cardiac troponin, myosin heavy chain, myosin-binding protein, and so on. Among those genes relevant to DCM, myocardial-related genes (*tnnc2*, *myhz1.1*, *tpm4b*, *myh6*, *myh7*, *myh7ba*, *scl8a1a*, *atp1b2a and cacng3b*) and cardiovascular development-related genes (*vegfc*, *nkx2.7*, *heg1*, *ccm2*, *ccm2l*, *klf2a*, *kdrl*, *id4* and *fosl2*) were revealed to be changed in NXT  +  TFD treated embryos compared with TFD-induced cardiomyopathy embryos as indicated by RNA-seq analysis. The expression pattern of the two group genes was clustered (Fig. 4A, B). qRT-PCR validation in zebrafish whole embryos further exhibited that the abnormal expression of these genes in TFD embryos was all significantly recovered to normal expression levels in varying degrees upon addition of NXT when compared with the expressions of these genes in control (Fig. 4C, D). This indicated that the therapeutic effect of NXT on DCM might be achieved by regulating genes related to myocardial and cardiovascular development.

**Table 1:** Zebrafish Homologues associated with human DCM genes based on RNA-seq data

Human DCM-associated gene	Zebrafish orthologue	Gene ID	Cardiomyopathy subtype(s)	log_2_(FoldChange)	padj
ACTC1 (cardiac actin)	actc1a	ENSDARG00000042535	DCM, HCM, LVNC	2.605409266	2.23E-07
	actc1b	ENSDARG00000076126	DCM, HCM, LVNC	3.204639486	7.79E-10
MYH6 (myosin heavy chain 6)	myh6	ENSDARG00000090637	DCM, HCM, CHD	2.966691137	4.27E-09
MYH7 (myosin heavy chain 7)	myh7	ENSDARG00000079564	DCM, HCM, LVNC	2.62564815	1.82E-07
	myh7ba	ENSDARG00000076075	DCM, HCM, LVNC	3.181888154	5.38E-10
	myh7bb	ENSDARG00000035322	DCM, HCM, LVNC	2.377845495	3.79E-06
	myh7l	ENSDARG00000079782	DCM, HCM	2.9740424	3.10E-09
	myhz1.1	ENSDARG00000067990	DCM, HCM	2.59151567	8.45E-05
TNNC (cardiac troponin C)	tnnc1a	ENSDARG00000011400	HCM, LVNC	2.529089309	6.54E-07
	tnnc1b	ENSDARG00000037539	HCM, LVNC	2.626641531	3.45E-07
TNNT (cardiac troponin T)	tnnt1	ENSDARG00000037954	DCM, HCM, LVNC	3.661572878	1.30E-05
	tnnt2a	ENSDARG00000020610	DCM, HCM, LVNC	2.345399238	3.72E-06
	tnnt2b	ENSDARG00000100694	DCM, HCM, LVNC	3.182087004	4.43E-04
TNNI (cardiac troponin I)	tnni4a	ENSDARG00000099870	DCM	4.604088258	7.20E-16
	tnni1b	ENSDARG00000052708	DCM, HCM	2.918318845	7.07E-09
	tnni3k	ENSDARG00000086933	DCM, HCM	2.40317011	5.28E-04
TTN (titin)	ttn.1	ENSDARG00000000563	DCM, HCM, ARVC	2.614507859	2.41E-07
	ttn.2	ENSDARG00000028213	DCM, HCM, ARVC	3.388962093	2.25E-11
MYBPC3 (myosin-binding protein C)	mybpc3	ENSDARG00000011615	DCM, HCM	3.171024301	3.74E-10
NEXN (nexilin)	nexn	ENSDARG00000057317	DCM, HCM	2.13189099	2.36E-04
NKX (NK2 homeobox)	nkx2.5	ENSDARG00000018004	DCM, HCM	2.318274388	4.34E-05
	nkx2.7	ENSDARG00000021232	DCM	2.074287056	4.63E-04
HEG1 (heart development protein with EGF like domains 1)	heg1	ENSDARG00000018441	DCM	3.092539	4.49E-09
TBX20 (T-box 20)	tbx20	ENSDARG00000005150	DCM	1.87288	4.85E-04
CCM2L (CCM2 like scaffold protein)	ccm2l	ENSDARG00000063089	DCM	2.074250424	2.49E-04
SLC8A1 (solute carrier family 8 member A1)	slc8a1a	ENSDARG00000013422	DCM, HCM	3.344777218	7.19E-11
LAMA2 (laminin, α2)	lama2	ENSDARG00000099390	DCM	1.937049252	3.66E-03
TPM4 (tropomyosin 4)	tpm4a	ENSDARG00000023963	DCM, HCM	2.275421133	7.24E-06
	tpm4b	ENSDARG00000019128	DCM, HCM	1.94540804	2.02E-04
ACTN2 (actinin, α2)	actn2b	ENSDARG00000071090	DCM, HCM	2.537681759	8.35E-07
CSRP3 (cysteine and glycine-rich protein 3)	csrp3	ENSDARG00000101706	DCM, HCM	5.082307638	5.42E-19
EYA4 (eyes absent homolog 4)	eya4	ENSDARG00000012397	DCM	1.763847085	2.92E-03

The fold change and adjusted *p* value were calculated by the R package DESeq in zebrafish embryonic heart treated with TFD over that treated with TFD  +  NXT, with |log_2_(FoldChange)|>  1 and padj  <  0.01

*DCM* dilated cardiomyopathy; *HCM* hypertrophic cardiomyopathy; *LVNC* left ventricular noncompaction; *RCM* restrictive cardiomyopathy; *ARVC* arrhythmogenic right ventricular cardiomyopathy; *CPVT* cathecolaminergic polymorphic ventricular tachycardia

### HEG1 signaling was one of targets of NXT in the treatment of cardiomyopathy

HEG1 (heart development protein with EGF-like domains 1) is a transmembrane receptor expressed in the endocardium, which mainly regulates adhesion between heart and vascular cells and regulates myocardial growth [30]. Studies in mice identified HEG1-CCM signaling as a critical regulator of cardiovascular organ formation and integrity [31]. In the RNA-seq data, *heg1* and *ccm* genes were screened as genes related to cardiovascular development in the zebrafish cardiomyopathy embryo heart when treated with NXT. It was suggested that HEG1-CCM signaling might be one of molecular targets of NXT in the treatment of cardiomyopathy.

**Fig. 5:**
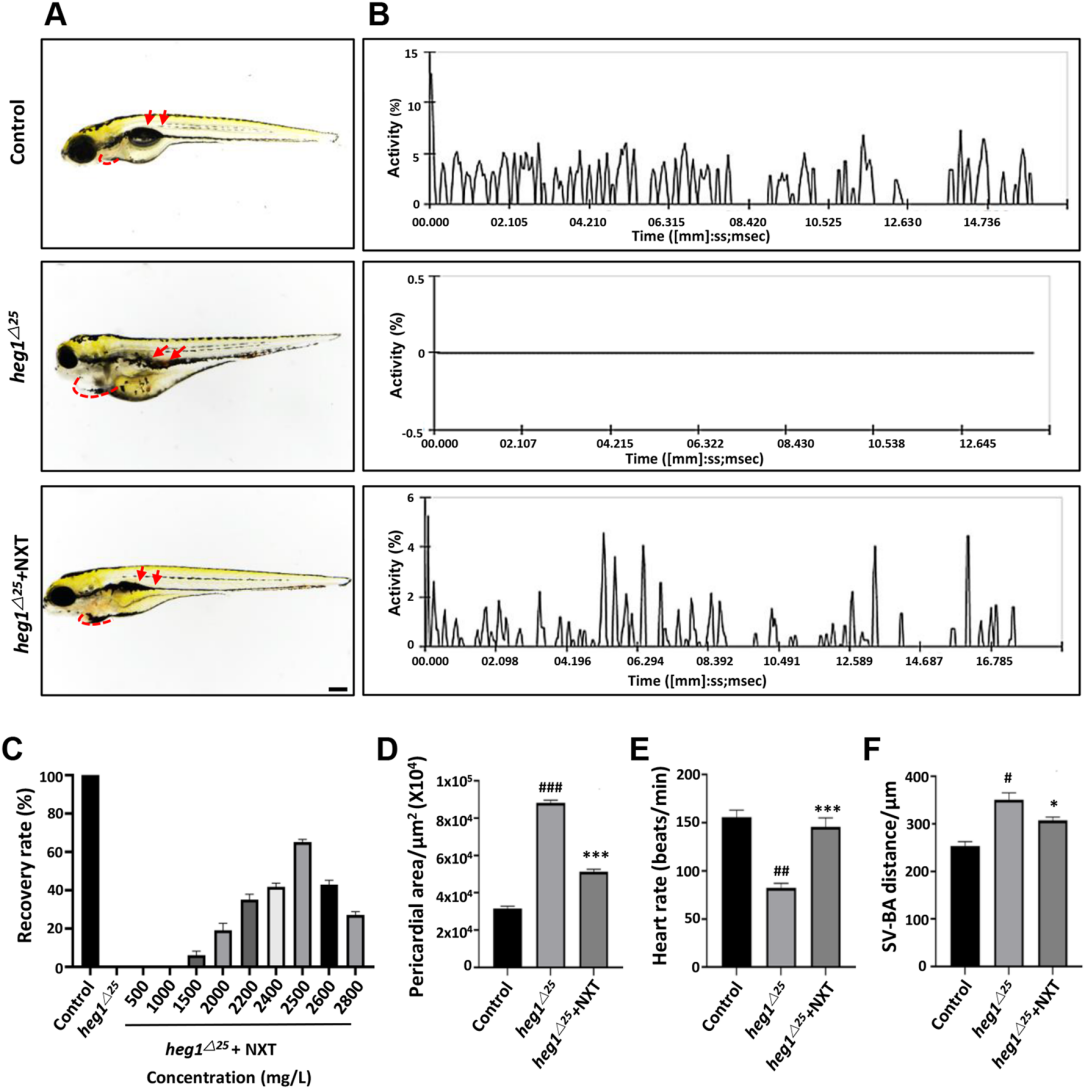
NXT restored cardiovascular malformation in *heg1*^*△25*^ mutant. **A** Lateral views of zebrafish embryos at 96 hpf. *heg1*^*△25*^ mutant embryos showing pericardial edema (red dashed line) and venous congestion (red arrows). **B** Blood flow motion ratios of three groups of zebrafish embryos based on RBCs pixel density changes. **C** Optimal recovery concentration of NXT. **D** Pericardial area of the three groups of zebrafish larvae (n  =  15 embryos/group). **E** Heart rates of three groups of zebrafish larvae (n  =  15 embryos/group). **F** SV-BA distance of three groups of zebrafish larvae (n  =  15 embryos/group). ^#^*p*  <  0.05, ^##^*p*  <  0.01, ^###^*p*  <  0.001 vs control group; *p  <  0.05, ***p  <  0.001 vs *heg1*^*△25*^ mutant group

**Fig. 6:**
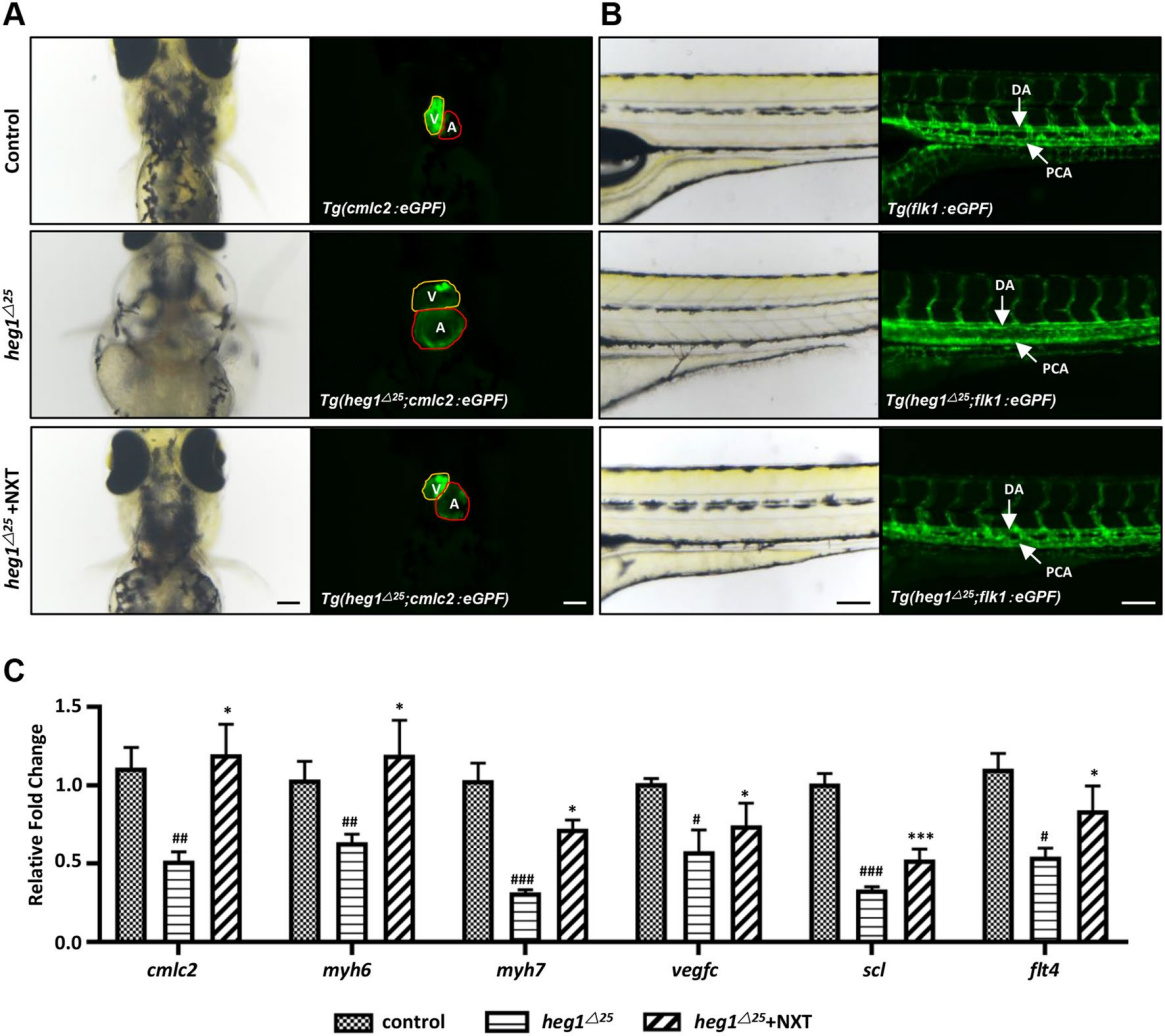
NXT ameliorated cardiovascular abnormalities in *heg1*^*△25*^ mutant. **A** Representative images of *Tg*(*cmlc2:eGFP*), *Tg*(*heg1*^*△25*^; *cmlc2:eGFP*) treated with or without NXT (ventral view). (*V* ventricle; *A* atria). **B** Representative images of *Tg*(*fik1:eGFPi*), *Tg*(*heg1*^*△25*^;* fik1:eGFP*) treated with or without NXT (lateral view). (*DA* dorsal aorta; *PCV* posterior main vein). **C** The expressions of cardiovascular markers, as determined by qRT-PCR, were significantly rescued by NXT treatment in *heg1*^*△25*^ mutant. Data are represented as mean  ± SD. ^#^*p*  <  0.05, ^##^*p*  <  0.01, ^###^*p*  <  0.001 vs control group; *p  <  0.05, ***p  <  0.001 vs *heg1*^*△25*^ mutant group

To further verify heg1 signaling, *heg1*^*△25*^ mutant, a congenital zebrafish cardiomyopathy model generated in our lab preciously [10] was treated with NXT. Similar to what observed in TFD-induced cardiomyopathy embryos, *heg1*^*△25*^ mutant embryo at 4 dpf was exhibited obvious pericardial swelling (Fig. 5A, D, red dotted line), blood flow stagnation and thromboembolism caused by cardiac pumping dysfunction (Fig. 5A, B), decreased heart rate (Fig. 5E), and abnormal increased SV-BA distance (Fig. 5F) when compared with control embryos. NXT was used to treat *heg1*^*△25*^ mutant at 2 dpf to find the optimal concentration for recovery (500 mg/L–2800 mg/L), and the phenotype was observed at 4 dpf (Fig. 5C). Under the treatment of 2500 mg/L NXT, those cardiovascular phenotypes of *heg1*^*△25*^ mutant were restored at various degrees, including pericardial area, blood flow, heart rate and SV-BA distance (Fig. 5A, B, D–F). To better observe the cardiovascular phenotype restoration of NXT in *heg1*^*△25*^ mutant, two *heg1* transgenic lines were used, *Tg*(*heg1*^*△25*^;* cmlc2:eGFP*) and *Tg*(*heg1*^*△25*^;* fik1:eGFP*) [10]. When treated with NXT, the enlarged heart chambers, especially atria, and coagulated blood vessels in *heg1*^*△25*^ mutant were significantly restored (Fig. 6A, B). Furthermore, the expression of myocardial tissue-specific markers, such as *cmlc2*, *myh6*, *myh7*, and the vascular markers such as *vegfc*, *scl*, *flt4* were abnormally decreased in *heg1*^*△25*^ mutant, which were restored to normal or near normal levels after treatment with NXT. These data suggested that the cardiovascular malformations of *heg1*^*△25*^ mutant could be restored under NXT treatment, and HEG1 signaling might be one of important molecular targets of NXT in the treatment of cardiomyopathy.

### Identification of NXT components by UPLC

**Fig. 7:**
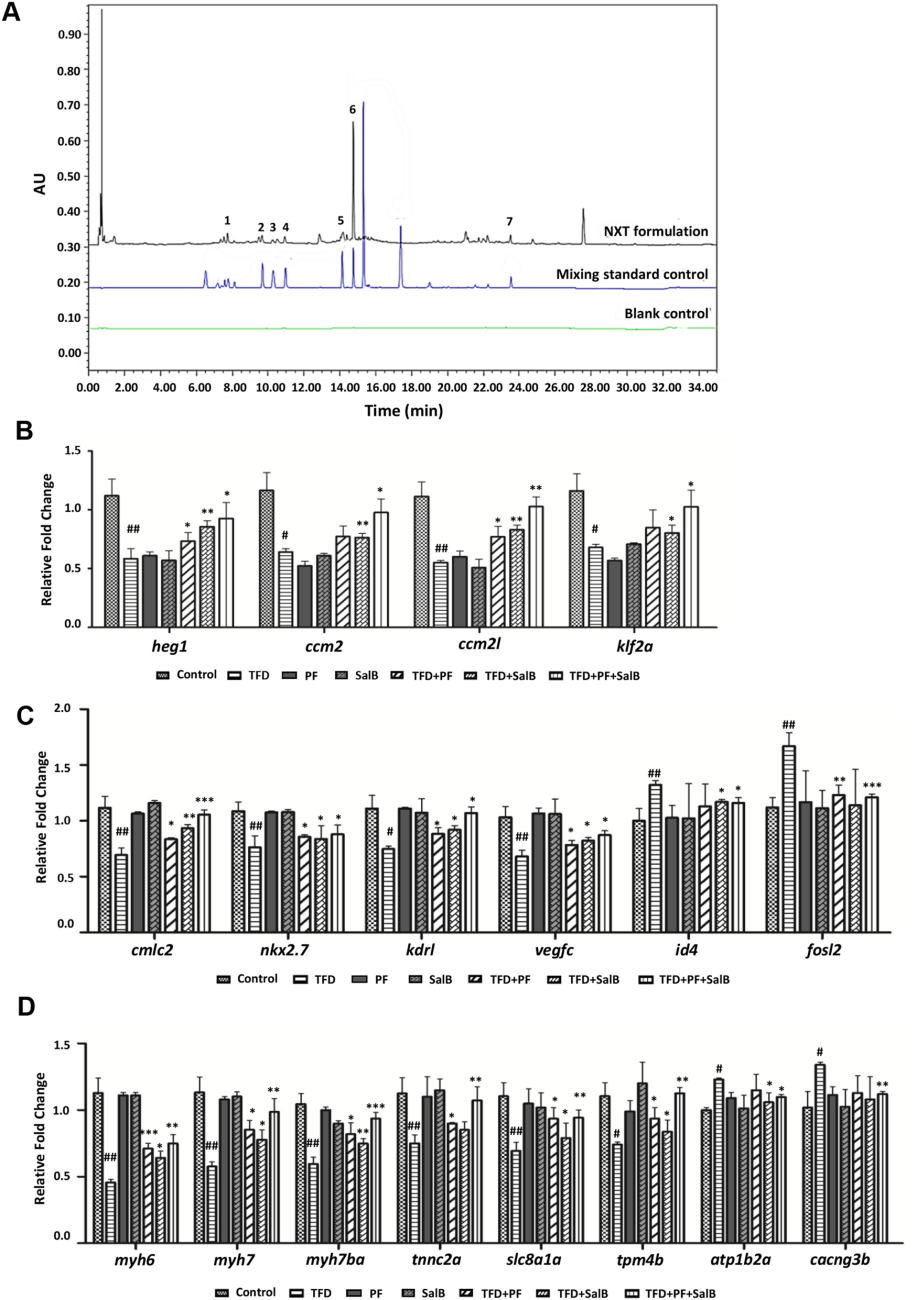
UPLC analysis of NXT combined with bioassays. **A** The fingerprints of the NXT. 1: HSYA, 2: PF, 3: FA, 4: CG, 5: RA, 6: Sal B, 7: Tan IIA. **B** HEG1-CCM signaling, **C** cardiovascular-related genes, **D** myocardial-related genes verified by qRT-PCR. Data are represented as mean  ±  SD. ^#^*p*  <  0.05, ^##^*p*  <  0.01, ^###^*p*  <  0.001 vs control group; *p  <  0.05, **p  <  0.01, ***p  <  0.001 vs TFD-induced group

As shown in Fig. 7A, there were seven major components in NXT identified as HSYA, PF, FA, CG, RA, Sal B, and Tan IIA, respectively. Among them, PF has vasodilatory and inhibitory effects on myocardial infarction and atherosclerosis [32]; Sal B can promote blood vessel growth, improve blood flow as well as prevent atherosclerosis [33]. The myocardial protective activity of PF and Sal B was investigated in TFD-induced zebrafish cardiomyopathy model by phenotypic examination (Additional file 4: Fig. S1) and qRT-PCR analysis (Fig. 7B–D). It indicated that PF and Sal B could significantly recovery cardiomyopathy phenotype (Additional file 4: Fig. S1), and restore abnormal expression of the vascular markers (Fig. 7C) and myocardial tissue-specific markers (Fig. 7D), as well as HEG1-CCM signaling (Fig. 7B).

## Discussion

Cardiomyopathy is a type of progressive heart disease in which the heart is abnormally enlarged, thickened, or rigid. It causes cardiac dysfunction due to damage to the myocardium and defective myocardial contractility. As a result, the heart becomes weaker and is less able to pump blood through the body, often causing heart failure and abnormal heart rhythms. Here, by using a TFD-induced zebrafish cardiomyopathy model, we were able to demonstrate that NXT could restore the reduced heart rate, the enlarged heart chambers, the reduced cardiac systolic function and the cardiac pumping dysfunction in zebrafish, indicating that NXT significantly improved cardiac dysfunction and had a good therapeutic effect on cardiomyopathy.

NXT has been approved by the Sino Food and Drug Administration (SFDA) (Z20025001) for clinical treatment of CVD. NXT had been shown to prevent atherosclerosis by improving blood lipid levels and restoring intestinal micro-ecological imbalance [20]. In a rat model of coronary heart disease with qi deficiency and blood stasis, NXT was found to regulate blood lipid levels, enhance antioxidant capacity, inhibit vascular inflammation and reduce myocardial damage, so as to inhibit the occurrence of coronary heart disease. The protective effect of NXT on myocardium in the treatment of coronary artery disease was mainly achieved by reducing the level of creatine kinase-MB [33]. A meta-analysis of randomized trials was performed in 2018, including 11 studies (N  =  1141) showing that NXT is an effective and safe therapy option for patients with cerebral infarction and carotid atherosclerosis [34]. However, the underlying molecular mechanism of NXT in the treatment of cardiomyopathy is still not well elucidated. In this study, zebrafish embryonic hearts were isolated from TFD treated group and TFD  +  NXT treated group, respectively, for transcriptome and bioinformatics analyses. A total of 961 DEGs was screened between TFD and TFD  +  NXT treated zebrafish hearts by RNA-seq analysis, including 441 up-regulated genes and 520 down-regulated genes. Furthermore, by comparing 30 DCM related genes homologous to human in zebrafish hearts based on RNA-Seq data, heg1 was identified as one of the important targets via which NXT could restore cardiomyopathy in zebrafish through HEG1-CCM signaling.

As a receptor, HEG1 usually binds to CCM1 and acts on heart and vascular development together with CCM1, CCM2 and PDCD10. The HEG1-CCM signaling pathway is crucial for cardiovascular development in zebrafish and mice [35]. In zebrafish, mutants *ccm1* (*santa*/*san*), *ccm2* (*valentine*/*vtn*), and *heg1* exhibited severe cardiac malformations and hemorrhagic arrest [30, 36]. In addition, knockdown of *ccm2l* (*ccm2*-like), an enhancer in the HEG1-CCM pathway, also caused dilatation of the atria and reduced or absent blood circulation in zebrafish [37]. RNA-seq analysis in this study revealed that heg1-ccm signaling was down-regulated in TFD-induced cardiomyopathy zebrafish embryonic hearts. However, the abnormal down-expressions of *heg1*, *ccm2*, and *ccm2l* in TFD-induced cardiomyopathy could be restored by NXT treatment confirmed by qRT-PCR validation. To further verify heg1 signaling regulated by NXT, *heg1*^*△25*^ mutant, a congenital DCM zebrafish model was applied to evaluate the therapeutic effect of NXT. The treatment of NXT on *heg1*^*△25*^ mutant not only significantly rescued the cardiomyopathy phenotypes, including the enlarged hearts and the stagnation of blood flow caused by cardiac pumping dysfunction, but also restored the abnormal expression of myocardial tissue-specific markers and vascular markers. These results suggested that NXT is a promising TCM compound for the treatment of cardiomyopathy.

Studies have shown that DCM is the result of multiple pathological mechanisms leading to cardiac dilation and associated cardiac dysfunction, with the changes in cardiac contractility and cardiac morphology accompanied by alterations in myocardial gene expression [38]. Through RNA-Seq screening of cardiomyopathy zebrafish embryonic heart treated with or without NXT, genes involved in myocardial contraction and myocardial tissue development were enriched. Among them, myosin heavy chain proteins (MYH) reflect the development of myofibrils and regulate cardiac contraction and cardiac physiological characteristics. There are nine MYH homologous genes in zebrafish [28]. Previous studies have shown that atrial myosin heavy chain (*myh6/amhc*) is specifically expressed in zebrafish atria, while ventricular myosin heavy chain (*myh7/vmhc*) is predominantly expressed in zebrafish ventricle [39]. Mutations of *myh6* and *myh7* in zebrafish are known to be associated with cardiomyopathy. The loss of myh6 function will impair atrial contractility and atrial muscle tubercle assembly, but will not affect ventricular contraction; while the loss of myh7 function will lead to excessive formation of myocytes and reduction of myogenic fibers in the ventricle [28, 40]. Therefore, *myh6* and *myh7* are important molecular biomarkers of zebrafish heart remodeling, and their expression directly affects the development and functional regulation of zebrafish heart. In the process of regulating myocardial contraction, cardiac troponin T (TNNT), cardiac troponin I (TNNI) and cardiac troponin C (TNNC) form troponin complexes, which are the preferred biomarkers of myocardial necrosis and provide a calcium-sensitive switch for myocardial contraction. TNNI serves to inhibit the interaction of actin and tropomyosin and to activate Ca^2+^ sensitivity through its interaction with TNNC. During contraction, calcium binds to TNNC, resulting in a conformational change that enhances the binding of TNNC to TNNI and regulates cardiac contraction [41]. In our study, the transcriptional expression of *myh6*, *myh7*, *myh7ba*, and *tnnc2* genes were significantly reduced in zebrafish embryos with cardiomyopathy, which directly affected myocardial structure formation, contraction and calcium ion regulation of zebrafish. Whereas NXT treatment could significantly restore the abnormal decreased expression of these genes, suggesting that NXT could promote the development of myofibril and the assembly of myosin, improve calcium ion sensitivity, and thus regulate myocardial contraction, not only in zebrafish, but also in humans.

## Conclusion

Through TFD-induced zebrafish cardiomyopathy model and the *heg1* mutant of the congenital zebrafish cardiomyopathy model, we proved that NXT had a good therapeutic effect on cardiomyopathy, and could restore myocardial injury and cardiac dysfunction. Transcriptome and bioinformatics analyses further revealed that NXT could restore cardiomyopathy in zebrafish through HEG1-CCM signaling, which might be one of molecular pathological mechanisms of NXT in the treatment of cardiovascular diseases. In addition, PF and Sal B could be considered as major bioactive components of NXT for myocardial protection in cardiomyopathy.

## Supplementary Information

**Additional file 1: Figure S1.** Restoration of TFD-induced zebrafish embryonic cardiomyopathy by PF and Sal B, two main components of NXT. **A** Lateral view of seven groups of zebrafish embryos at 4 dpf. TFD treated group showing pericardial edema (red dashed line) and slowed blood flow (red arrows). **B** Blood flow motion ratios of seven groups of zebrafish embryos based on pixel density changes in RBCs. **C** Heart rates of seven groups of zebrafish larvae (n=15 embryos/group). **D** Pericardial area of seven groups of zebrafish larvae (n=15 embryos/group). **E** SV-BA distance of the seven groups of zebrafish larvae (n=15 embryos/group). Data are represented mean ± standard deviation (SD) from three independent experiments, ^###^*p *< 0.001 vs control group; **p < 0.01, ***p < 0.001 vs TFD-induced group.

**Additional file 2: Table S1.** List of all primer sequences.

**Additional file 3: Table S2. **DGEs of TFD+NXT vs. TFD.

**Additional file 4: Table S3.** GO and KEGG analysis of DGEs of TFD+NXT vs. TFD.

## Data Availability

The research data generated from this study is included within the article.

## Abbreviations

NXT: Naoxintong
TCM: Traditional Chinese Medicine
TFD: Terfenadine
CVD: Cardiovascular diseases
HCM: Hypertrophic cardiomyopathy
DCM: Dilated cardiomyopathy
RCM: Restrictive cardiomyopathy
ARVC: Arrhythmogenic right ventricular cardiomyopathy
SV-BA: Sinus venosus and bulbus arteriosus
I/R: Ischemia/reperfusion
RBCs: Red blood cells
PVCs: Posterior cardiac veins
qRT-PCR: Real-time quantitative PCR
UPLC: Ultra performance liquid chromatography
MulA: Mulberroside A
HSYA: Hydroxysafflor yellow A
AMY: Amygdalin
PF: Paeoniflorin
FA: Ferulic acid
CG: Calycosin 7-*O*-glucoside
RA: Rosmarinic acid
Sal B: Salvianolic acid B
CAL: Calycosin
FN: Formononetin
Tan IIA: Tanshinone IIA
GO: Gene ontology
HEG1: Heart development protein with EGF-like domains 1
MYH: Myosin heavy chain proteins
TNNT: Cardiac troponin T
TNNI: Cardiac troponin I
TNNC: Cardiac troponin C
