# Supplementary figures and images for: Myocardial protective effect and transcriptome profiling of Naoxintong on cardiomyopathy in zebrafish
Source: Chin Med. 2021 Nov 14;16:119. doi: 10.1186/s13020-021-00532-0 (PMC8591872; doi:10.1186/s13020-021-00532-0)

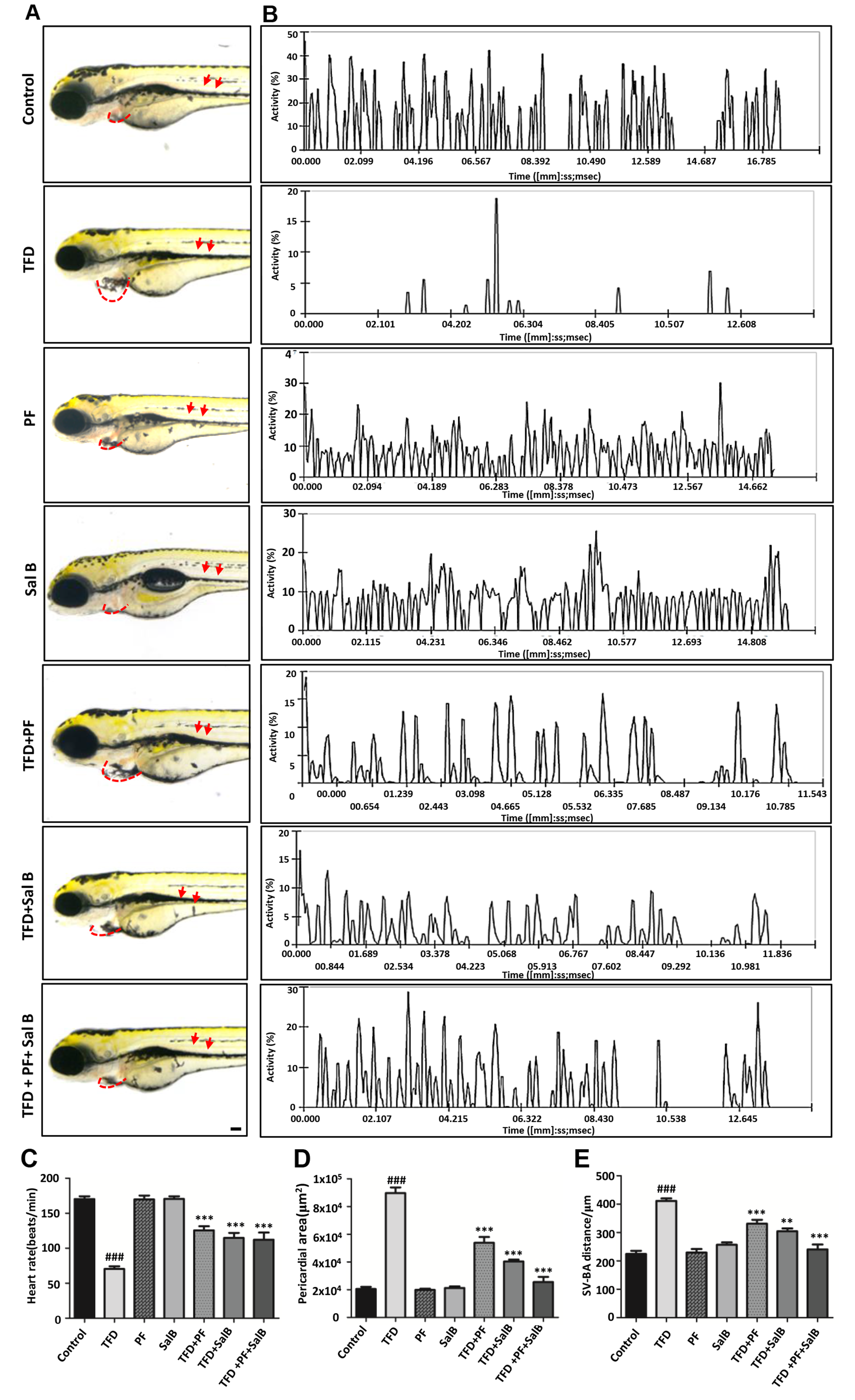

Supplement: Supplementary file 1 — Additional file 1: Figure S1. Restoration of TFD-induced zebrafish embryonic cardiomyopathy by PF and Sal B, two main components of NXT. A Lateral view of seven groups of zebrafish embryos at 4 dpf. TFD treated group showing pericardial edema (red dashed line) and slowed blood flow (red arrows). B Blood flow motion ratios of seven groups of zebrafish embryos based on pixel density changes in RBCs. C Heart rates of seven groups of zebrafish larvae (n=15 embryos/group). D Pericardial area of seven groups of zebrafish larvae (n=15 embryos/group). E SV-BA distance of the seven groups of zebrafish larvae (n=15 embryos/group). Data are represented mean ± standard deviation (SD) from three independent experiments, ###p < 0.001 vs control group; **p < 0.01, ***p < 0.001 vs TFD-induced group. [file 13020_2021_532_MOESM1_ESM.tif]
