# Supplementary material for: Myocardial protective effect and transcriptome profiling of Naoxintong on cardiomyopathy in zebrafish
Source: Chin Med. 2021 Nov 14;16:119. doi: 10.1186/s13020-021-00532-0 (PMC8591872; doi:10.1186/s13020-021-00532-0)
Supplement: Supplementary file 2 — Additional file 2: Table S1. List of all primer sequences. [file 13020_2021_532_MOESM2_ESM.docx]

**Supplementary Table S1: List of all primer sequences**

Zebrafish cDNA primer sequence used for Q-PCR

| Gene | Primer | Sequence |
| --- | --- | --- |
| *myh7*  *slc8a1a*  *tnnc2*  *tpm4b*  *myh7ba*  *ccm1*  *ccm2*  *ccm2l*  *klf2a*  *kdrl*  *flt4*  *scl*  *vegfc*  *nkx2.7*  *myhz1.1*  *heg1*  *myh6*  *gapdh* | forward  reverse  forward  reverse  forward  reverse  forward  reverse  forward  reverse  forward  reverse  forward  reverse  forward  reverse  forward  reverse  forward  reverse  forward  reverse  forward  reverse  forward  reverse  forward  reverse  forward  reverse  forward  reverse  forward  reverse  forward  reverse | TCAGATGGCAGAGTTTGGAG  GCTTCCTTTACAGTTACAGTCTTTC  ATACCTCACTGGGAACATG  CATTCATAACTACCACCACAT  ACTGACGCGCAACAGGA  CTGCCGTCTTCATCGACCT  ACAAACAGCTGGAAAACGAG  TCCTGAATTTCCATTTTCTCC  GCCTGAACTTCTTGACATGC  CTCCCTCTGCTTCTGTTTGA  CATAATAGGGAAGCGTGTTGTG  GGAGGAGAAATGAGCACTGG  CGTCTATACCGAGTCCACCA  AGGAGTCTTCACTGTAGATTGAG  AGGTCAAGTTCCTGGGACAC  CAGACAGACTGAGAATACAGTCC  CTGGGAGAACAGGTGGAAGGA  CCAGTATAAACTCCAGATCCAGG  GACCATAAAACAAGTGAGGCAGAAG  CTCCTGGTTTGACAGAGCGATA  ATTACAACTGCGTGCCGTTT  TGTCAACATGGCTCCTCTGT  GCCAATGGTGAAGTTGTGAGT  CGTCTGCTCTCTACCTGGAT  AGTTCCAGTCAAGCAAGGGA  TGAATGAAGGGTGTCAGGCA  CGGAGAACAGGTGAAATGCG  TGTAACTGGAGTCGGTAAATGG  AGCTGATACGTCAAGGGAACA  CAGAACAGCCCAGAGTAGGT  CCACTGCCACAGCCGTGGATC  GTCAGATTGAAGATGTTCTG  GACATGGCGATGCTGACGTTTC  GATAAGCATTATCTGAGATG  CCAAGGCTGTAGGCAAAGTAA  AAGATGGATGAACGGCAATC |
